# Immune responses in liver and spleen against *Plasmodium yoelii* pre-erythrocytic stages in Swiss mice model

**DOI:** 10.1016/j.jare.2020.02.016

**Published:** 2020-02-26

**Authors:** Arif Jamal Siddiqui, Jyoti Bhardwaj, Manish Goyal, Kirtika Prakash, Mohd Adnan, Mousa M. Alreshidi, Mitesh Patel, Awakash Soni, Whitni Redman

**Affiliations:** a Department of Biology, College of Sciences, University of Ha’il, Ha’il, Saudi Arabia; b Division of Parasitology, CSIR-Central Drug Research Institute, Lucknow, Uttar Pradesh, India; c Indiana University, School of Medicine, Indianapolis, IN, United States; d Department of Obstetrics, Gynecology and Reproductive Sciences, College of Medicine, University of Vermont, VT, United States; e Bapalal Vaidya Botanical Research Centre, Department of Biosciences, Veer Narmad South Gujarat University, Surat, Gujarat, India; f Surgery Department, Division of Biomedical Research, Texas Tech University Health Sciences Center, Lubbock, TX, United States

**Keywords:** *Plasmodium yoelii*, Swiss mice, Pre-erythrocytic stage, Immune responses, Splenic cells, T cells

## Abstract

Though the immunity to malaria has been associated with cellular immune responses, the exact function of the phenotypic cell population is still unclear. This study investigated the host immune responses elicited during the pre-erythrocytic stage, post-*Plasmodium yoelii* sporozoite infection in Swiss mice model. For this purpose, we analyzed the dynamics of different subsets of immune cells population and cytokine levels in the hepatic mononuclear and splenic cells population during pre-erythrocytic liver-stage infection. We observed a significant reduction in the effectors immune cells population including CD8^+^ T cell, F4/80^+^ macrophage and in plasmacytoid dendritic cells (CD11c^+^ B220^+^). Interestingly, substantial down-regulation was also noted in pro-inflammatory cytokines (i.e. IFN-γ, TNF-α, IL-12, IL-2, IL-17 and iNOS), while, up-regulation of anti-inflammatory cytokines (i.e. IL-10, IL-4 and TGF-β) during asymptomatic pre-erythrocytic liver-stage infection. Collectively, this study demonstrated that during pre-erythrocytic development, *Plasmodium yoelii* sporozoite impaired the host activators of innate and adaptive immune responses by regulating the immune effector cells, gene expression and cytokines levels for the establishment of infection and subsequent development in the liver and spleen. The results in this study provided a better understanding of the events leading to malarial infection and will be helpful in supportive treatment and vaccine development strategy.

## Graphical abstract

**Figure:**
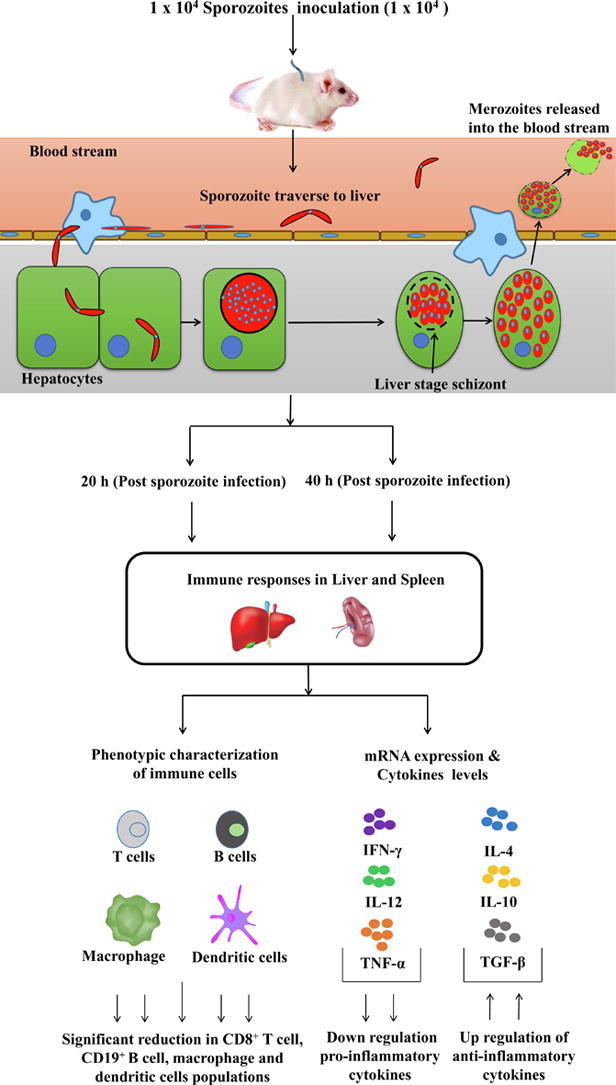

## Introduction

Malaria is one of the highly fatal infectious diseases that cause more than 400,000 casualties and ~200 million new cases, annually [1]. In humans, malaria infection begins when malaria-infected female *Anopheles* mosquito bites and released its sporozoites into the bloodstream of the human host. Once enters the bloodstream, the sporozoites quickly travel to the liver and infects the hepatocytes. Inside the hepatocyte, sporozoites multiply asexually and ultimately release thousands of merozoites into the bloodstream [2], [3]. These merozoites invade the erythrocytes and initiate the asexual blood-stage cycle (intra-erythrocytic stages), which is responsible for the clinical manifestation of malaria [4], [5]. As a response to malarial infection antiparasite immune responses gets activated that can efficiently control malaria parasite infection at all development stages i.e. pre-erythrocytic, asexual stages in red cells, and sexual and mosquito stages. The role of the innate immune responses by eliciting the pro-inflammatory cytokines such as interferon γ (IFNγ), tumor necrosis factor α (TNFα) and interleukin-12 (IL12) during blood-stage infection was well documented. However, our current knowledge regarding the innate immune responses generated during the pre-erythrocytic stages is very little.

It has been suggested that in liver innate immune responses can impede parasites and cytokines released from innate immune cells may help in the shaping of the adaptive antiparasitic immune responses [6], [7]. However, recognition of the liver stage antigens by innate immune cells and host defense against the liver-stage parasite still remains unanswered. Apart from this, the availability and function of phenotypic cells population governing the host immune responses towards the infection are also less understood. Studies in rodent malaria indicate that both murine CD8^+^ and CD4^+^ T cells can target pre-erythrocytic parasite stages within the liver [8]. It has been observed that inhibition of pre-erythrocytic stage development was handled by IFN-γ produced by both CD4^+^ and CD8^+^ T cells [9], [10]. Besides this, studies also reported the infiltration of eosinophils and neutrophils during liver-stage development [8]. Furthermore, studies also documented the role of dendritic cells (DCs) in sporozoite-induced protective immunity [11]. Phenotypically, diverse subsets of DCs have rare phenotypes with various immune functions and are identified as major histocompatibility class II (MHCII) molecules [11]. In this view, the role and function of DCs subsets during pre-erythrocytic stage development is very crucial. On the other hand, the malaria parasite also modulates the host immune cells to establish the infection. One of such example is Kupffer cells (KCs), which possess a broad range of class I, class II, co-stimulatory and adhesion molecules that help in boosting cytokine production and T cell multiplication [12]. It has been observed that sporozoites can manipulate the KCs function by negatively affecting their antigen-presentation capacity and modulating cytokine profile [13], [14]. Altogether these studies advocate the importance of studying the cellular immune responses and the role of diverse phenotypic cells population during the pre-erythrocytic *Plasmodium* infection.

The present study is an attempt to answer these questions employing sporozoite induced *Plasmodium yoelii* (*P. yoelii*) infection in Swiss mice model. We have explored various immune responses generated during primary sporozoite infection in mice. Specifically, we have determined the mRNA expression profile of different cytokines along with the dynamics of different immune cell populations (including T cells, B cells, macrophage and subset of DCs in liver and spleen of mice, following sporozoite infection). In order to correlate immune responses with liver-stage parasite growth, real-time PCR based protocol for quantitative assessment of liver-stage parasite burden in the host was determined.

## Materials and methods

### Ethics statement

All efforts were made to curtail the suffering throughout this study. Therefore, all mice were sacrificed using deep ether anesthesia. Ethical approval (No: IAEC/2012/67-N) was obtained from CSIR-Central Drug Research Institute’s ‘Institutional Animal Care and Use Committee’ recognized by ‘Committee for the Purpose of Control and Supervision of Experiments on Animals (CPCSEA)’, Government of India to perform experiments on animals.

### In vivo malaria model

6–8 weeks old laboratory-bred free female Swiss Albino mice (weight 22–24 g) were used for all the experiments in accordance with our Institutional Animal Ethics Committee (IAEC) guidelines. Maintenance of the mice and parasitic cycle at the institute’s laboratory animal facility has been described and detailed previously [2], [15], [16]. In brief, the parasite cycle was routinely maintained by the alternating passage of blood-stage parasite (1 × 10^6^) in Swiss Albino mice. Sporozoites were isolated from *Anopheles stephensi* mosquitoes salivary glands at day 12 following infectious blood meal (mice) and isolated as described earlier with minor modifications [5]. Salivary glands were dissected from infected or uninfected mosquitoes and ground with a probe in a small volume of RPMI 1640 medium followed by centrifugation at 1000 rpm for 1 min at 4 °C. The resulting supernatant was collected in a separate tube and observed under a phase-contrast microscope for the presence of sporozoites. The sporozoite numbers were counted by hemocytometer. Mice were injected intravenously either with 1 × 10^4^ sporozoites or salivary gland debris from uninfected mosquitoes as control. During the pre-erythrocytic stage (stage followed by sporozoite inoculation) symptoms of severe disease were absent because the pre-erythrocytic stage is asymptomatic (clinically silent). But once parasite enters into blood-stage the symptoms of the disease begin to appear (i.e. fever, weight loss, fatigue, and anemia) and mice were died after day 12 due to severe anemia and multi-organ failure.

### Assessment of liver and blood-stage parasite burden

Real-time PCR was used to determine the liver stage parasite burden following *P. yoelii* sporozoite inoculation in mice as described previously [15], [16], [17]. In brief, the liver was individually collected from control uninfected (n = 5) or sporozoite infected mice at 20 h (n = 5) and 40 h (n = 5) post sporozoites inoculation and homogenized in 10 ml Trizol reagent (Thermo Fisher, Waltham, Massachusetts, USA). The total RNA from the liver of infected and uninfected mice was isolated, quality checked, quantified, and converted to cDNA and stored at −20 °C as described previously [15], [16], [17]. The liver stage parasite burden was quantified by determining the *P. yoelii* specific 18S rRNA copy number in control and infected mouse liver. For the blood-stage parasite burden, the mice from control uninfected (n = 5) and infected group (n = 5) were checked daily for blood-stage patency by Giemsa staining of thin blood smears.

### Hepatic mononuclear cells (HMNCs) and splenic cells isolation

To characterize the immune responses in the liver and spleen against the pre-erythrocyte stage mice were injected either with 1 × 10^4^ sporozoites or untreated as a control. The liver and spleen were individually collected from control uninfected (n = 6) or sporozoite infected mice at 20 h (n = 6) and 40 h (n = 6) post sporozoites inoculation. The liver cell suspension was prepared by perfusion of the liver with 1X PBS as described previously with some modifications [18]. Swiss mice were anesthetized with chloroform, and their abdomens were opened, and a needle was inserted into the portal vein. The inferior vena cava was cut to enable blood outflow to rule out the contamination of peripheral blood monocytes and the liver was perfused with 20 ml of 1X PBS (pH 7.2). Following perfusion liver was gently meshed on a strainer to prepare a single-cell suspension. Subsequently, the liver cell suspension was centrifuged at 85X g for 1 min at room temperature and the supernatant was carefully transferred into a new tube. The supernatant was again centrifuged at 450X g for 5 mins at room temperature. The supernatant was then discarded and the cell pellet was re-suspended in 6 ml of 40% Percoll (Sigma, St Louis, Missouri, USA). It was then carefully overlaid on 3 ml of 70% Percoll and centrifuged at 800X g for 20 mins at room temperature to prepare gradient. The middle layer comprising of leukocytes was carefully taken out and transferred to a new tube. Two volumes of 1X PBS (pH 7.2) was added and again centrifuged on 800X g for 5 mins at room temperature. The supernatant was discarded and the cell pellet finally re-suspended in 3 ml of complete RPMI 1640 medium.

For splenic cells isolation**,** the individual spleen was aseptically removed and crushed on a 70 µm cell strainer to prepare the single-cell suspension in incomplete RPMI 1640. The suspension was centrifuged at 450X g for 5 mins and the pellet was re-suspended in 1X RBC lysis buffer (BD bioscience, Qume Drive, San Jose, USA) and incubated for 2 mins at 37 °C in CO_2_ incubator. Cells were washed with 1X PBS (pH 7.2) and pellet down at 450X g for 5 mins and re-suspended in 5 ml of complete RPMI 1640 medium. Cells were counted using a hemocytometer and the cell viability checked by the Trypan Blue Exclusion method. The isolated HMNCs and splenic cells were further used for cell culture, phenotypic analysis, and mRNA expression analysis of different cytokines.

### Immunophenotypic analysis of immune cells by flow cytometry

The phenotypic characterization of HMNCs and splenic cells was performed by flow cytometry using specific fluorochrome-conjugated antibodies such as FITC-anti-CD4 (Helper T cell), V450-anti-CD8 (Cytotoxic T cell), PE-anti-CD19 (B cell), PerCP Cy5.5-anti-CD11c (Dendritic cell), PE Cy7-anti-CD11b (Monocytes cell), PerCPCy5.5-anti-B220/CD45R, APC-anti-NK1.1 (Natural Killer cell), and APC-anti-F4/80 (Macrophage) antibodies. Furthermore, we characterization of sub-lineages of plasmacytoid DCs, based on the expression of CD8, CD4 and CD11b surface markers, the following six distinct subsets were defined within the NK1.1^−^ CD11c^+^ B220^+^ HMNDC population which are NK1.1^−^ CD11c^+^ B220^+^ CD8^+^, NK1.1^−^ CD11c^+^ B220^+^ CD8^−^, NK1.1^−^ CD11c^+^ B220^+^ CD4^+^, NK1.1^−^ CD11c^+^ B220^+^ CD4^−^, NK1.1^−^ CD11c^+^ B220^+^ CD11b^+^, and NK1.1^−^ CD11c^+^ B220^+^ CD11b^−^. All antibodies were purchased from BD Bioscience (Qume Drive, San Jose, USA). Briefly, cells were incubated with Fc blocker (CD16/32) for 15–20 mins at 4 °C in dark to avoid non-specific binding. After 20 mins, cells were washed with 1X PBS and centrifuged at 450X g for 5 mins and then stained with a specific fluorochrome-conjugated antibody for 20 mins in dark at 4 °C. Following antibody staining, cells were washed and re-suspended in 300 µl 1X PBS supplemented with 2% FBS for FACS analysis. Flow cytometry analysis of different cell populations was performed on a FACS Aria (BD Biosciences, Qume Drive, San Jose, United State) flow cytometer and data were analyzed using FlowJo 8.1.0 software (Cell population).

### Real-time PCR for mRNA expression of cytokines

The mRNA expression of different cytokines in HMNCs and splenocytes (isolated from mice of different groups) was determined using real-time PCR. Briefly, total RNA was extracted from HMNCs and splenocytes using the Qiagen RNA kit (Cat no.74106, Qiagen Hilden, Germany). The extracted RNA was used for cDNA synthesis and served as a template for real-time PCR [16]. The primers for mRNA expression of various cytokines were designed using Gene Runner version 3.05 using the gene sequences of mice available in NCBI gene bank as listed in Table 1. All real-time PCR data was acquired on the Roche® light cycler 480.0 instrument and analyzed using Roche-Gene software version 1.5.0.

**Table 1:** Primer sequences used for quantitative RT-PCR to determine *P. yoelii* liver stage parasite load and mRNA expression level of different cytokines.

Gene	Primer sequence	
**18S rRNA**	Forward	5′GGGGATTGGTTTTGACGTTTTTGCG 3′
	Reverse	5′AAGCATTAAATAAAGCGAATACATCCTTAT 3′

**GAPDH**	Forward	5′ ACAGTCAAGGCCGAGAATGGG 3′
	Reverse	5′GCCGGTGCTGAGTATGTCGT 3′

**IFN-γ**	Forward	5′ GTTACTGCCACGGCACAGTCATTG 3′
	Reverse	5′ACCATCCTTTTGCCAGTTCCTCCAG 3′

**TNF-α**	Forward	5′ CCGATGGGTTGTACCTTGTCT 3′
	Reverse	5′GTGGGTGAGGAGCACGTAGT 3′

**IL-12**	Forward	5′ GGAAGCACGGCAGCAGAATA 3′
	Reverse	5′AACTTGAGGGAGAAGTAGGAATGG 3′

**iNOS**	Forward	5′ TCCTCACTGGGACAGCACAGAATG 3′
	Reverse	5′ GTGTCATGCAAAATCTCTCCACTGCC 3′

**IL-10**	Forward	5′ TTTGAATTCCCTGGGTGAGAA 3′
	Reverse	5′ACAGGGGAGAAATCGATGACA 3′

**IL-4**	Forward	5′ TCAACCCCCAGCTAGTTGTC 3′
	Reverse	5′TCTGTGGTGTTCTTCGTTGC 3′

**IL-13**	Forward	5′ TGCGGTTACAGGCCATGCAATA 3′
	Reverse	5′TGAGGAGCTGAGCAACATCAA 3′

**TGF-β**	Forward	5′ AACTATTGCTTCAGCTCCACAG 3′
	Reverse	5′AGTTGGCATGGTAGCCCTTG 3′

### Culture of isolated hepatic mononuclear cells and splenocytes and cytokines analysis

Isolated HMNCs and splenocytes from the respective samples were seeded into 48-well tissue culture plates (0.5 × 10^6^ cells per well) in triplicates and incubated at 37 °C in a CO_2_ incubator for 24 h to allow the cell adherence. After 24 h, fresh complete RPMI 1640 medium was added to each well and cells were stimulated with either lipopolysaccharides (LPS) (1 µg/well for nitric oxide (NO) and cytokine assays) or concanavalin A (5 µg/well for cell proliferation assay) and further incubated for 48 h at 37 °C in CO_2_ incubator. Subsequently, supernatants were collected from LPS stimulated cells and used for nitric oxide and cytokine estimation. In addition, XTT cell proliferation reagent (Sigma®, St Louis, Missouri, USA) was added to Concanavalin A (ConA) stimulated cells to analyze the cell proliferation response.

### Assay for nitric oxide

NO released in culture supernatants of hepatic and splenic macrophages was determined by Griess reagent (Sigma®, St Louis, Missouri, USA). Griess reagent was added into culture supernatants in a 1:1 ratio and further incubated for 15 mins. After the incubation period, optical density at 540 nm was measured using ELISA plate reader. NO concentration in each culture supernatant was calculated by using a standard curve derived from ten-fold serial dilutions of known concentration of sodium nitrite and its corresponding absorbance value using a linear regression equation.

### Estimation of cytokine concentration in cell supernatants

Cytometric Bead Array (CBA) was used for determining the cytokine concentration (Th1, Th2, and Th17) in the culture supernatants of hepatic and splenic macrophages from different groups of mice. Mouse Th1/Th2/Th17 cytokine kit (BD Bioscience, Qume Drive, San Jose, USA) was used following the manufacturer’s protocol. Samples were acquired on BD FACS Calibur flow cytometer equipped with a blue and red laser. For data analysis, FCAP Array software (BD Bioscience, Qume Drive, San Jose, USA) was used.

### Assay for cell proliferation

To check the cell proliferation, XTT cell proliferation assay kit (Sigma®, St Louis, Missouri, USA) was used. This kit measures the cell proliferation based upon the reduction of the tetrazolium salt, 2,3-Bis (2-methoxy-4-nitro-5-sulfophenyl)-2H-tetrazolium-5-carboxanilide [19]. After Concanavalin A (ConA) stimulation for 48 h, XTT reagent was added in each well and cells were further incubated for 20 h at 37 °C in CO_2_ incubator. After incubation, the plate was gently shaken on the rocker to evenly distribute the dye in the wells. Using ELISA plate reader, absorbance was measured at 450 nm considering 650 nm wavelength as reference.

### Statistical analysis

All statistical analysis was performed using GraphPad Prism 5.0 Software, GraphPad Software, La Jolla California USA, (www.graphpad.com) [20]. Statistical significance was determined using Student’s *t*-test. *P* values < 0.05 were considered significant [21].

## Results

### Determination of liver-stage parasite burden following post sporozoite infection

**Fig. 1:**
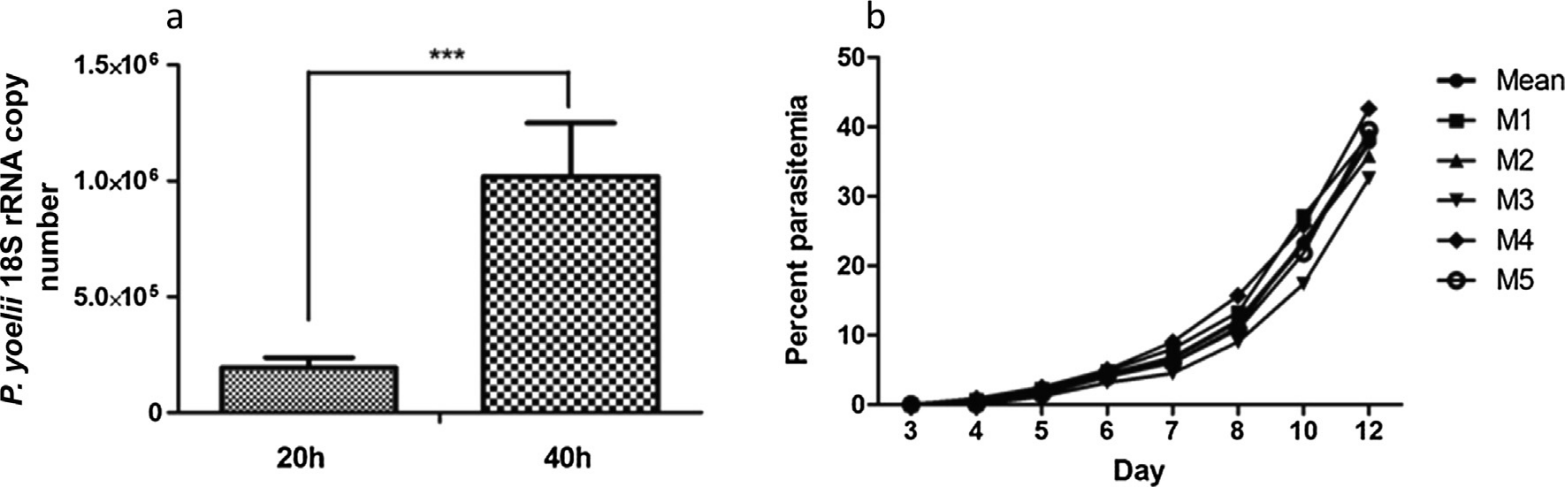
Quantification of liver-stage and blood-stage parasite load following *P. yoelii* sporozoite infection. (a) Relative expression levels of Py18S rRNA normalized to the mouse GAPDH. The bar graph represents Mean ± SD (n = 5) Py18S rRNA copy number. Statistical significance between control and sporozoite infected mice was determined using the student’s *t*-test (*** = p < 0.001). (b) Blood stage patency in sporozoite infected mice. The line graph shows the day-wise blood-stage parasitemia of individual mice from the sporozoite infected group.

The Liver stage or pre-erythrocytic stage is the first step of malaria infection that lies between sporozoite invasion of hepatocytes and merozoites released from the liver. The hepatic stage is clinically silent but represents a potential site to initiate the systematic immune responses to control the parasite. In this view, we first injected the mice with live sporozoite and then follow their subsequent development in the liver by measuring the liver stage parasite load at different time points post sporozoite injection i.e. 20 h and 40 h. To detect the liver stage parasite load we used a robust quantitative RT-PCR based system to detect 18S rRNA copy number as described previously [15]. Our results revealed that at 20 h post sporozoite infection 18S rRNA copy number was 193417.6 ± 42832.5, which was further increased to 1018709.0 ± 229151.3 at 40 h post sporozoite infection. This showed an almost 5 fold increase in the 18S rRNA copy number between the 20 h and 40 h post sporozoite inoculation period, suggesting rapid development of the pre-erythrocytic stages during this time (Fig. 1a). Apart from this, we also checked mice for intraerythrocytic blood-stage parasite load. For this purpose, the course of blood infection was recorded in control and infected mice (n = 5) from day 1 post sporozoite inoculation. The results showed that all the infected mice developed patency on day 4 and followed the usual course of infection, while control mice were negative for blood-stage infection (Fig. 1b). In short, our results validated that the sporozoites used in this study were infective/ viable and at an initial 40 h post sporozoite infection only pre-erythrocytic stage present, while blood-stage developed later on (Fig. 1a and Fig. 1b).

### Dynamics of cellular immune responses elicited during pre-erythrocytic stage development

#### Assessment of T, B lymphocytes and macrophages in HMNCs

**Fig. 2:**
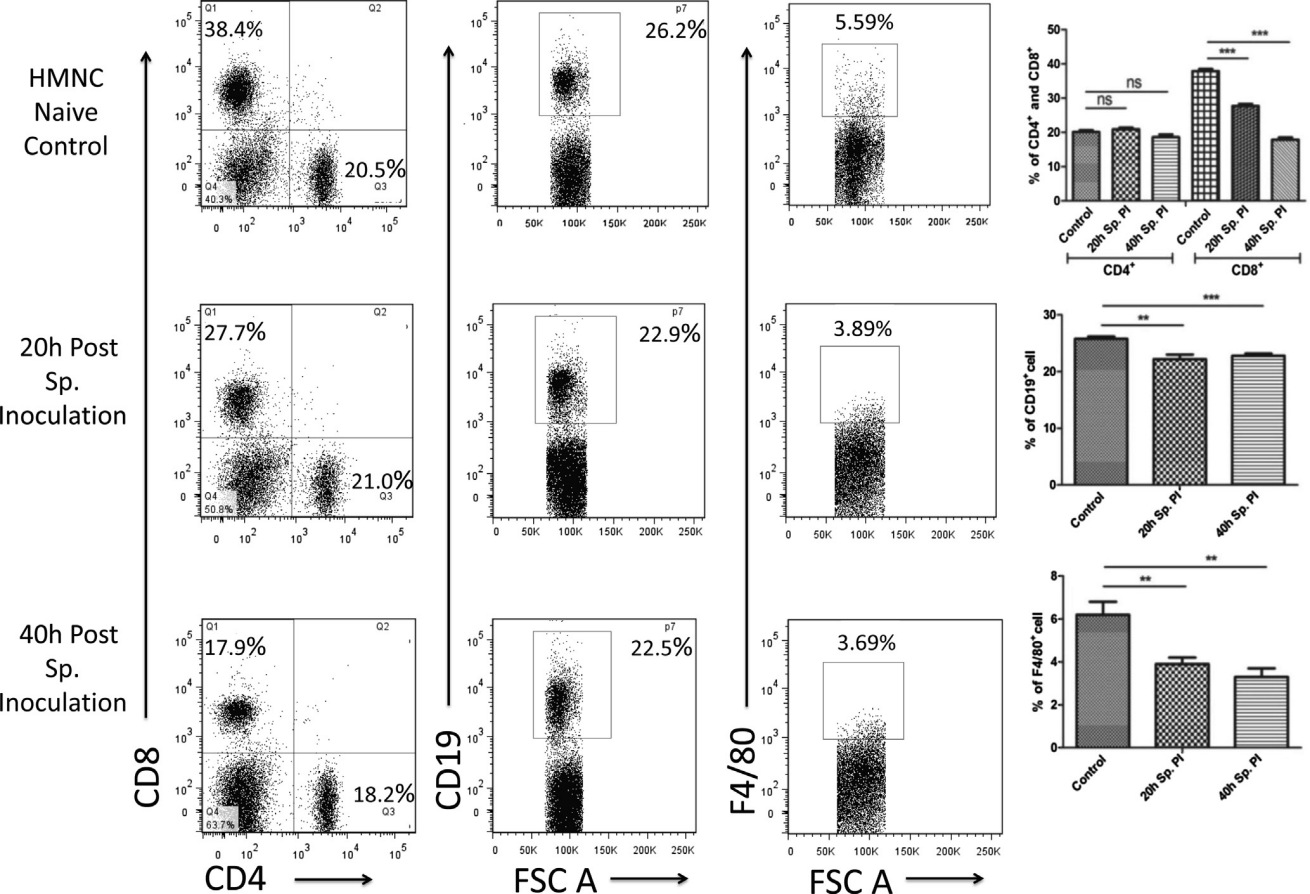
Phenotypic analysis of T cell, B cell and macrophage populations in HMNCs after *P. yoelii* sporozoite induced infection in mice. Percentage CD4^+^ and CD8^+^ population were determined in HMNCs isolated from different groups (naive and sporozoite infected mice) using flow cytometry. Dot plots show representative images from one experiment and show the percentage population of CD4^+^, CD8^+^, CD19^+,^ and F4/80^+^ cell populations, while the bar graph shows the Mean ± SD percentage population (n = 6). Statistical significance between different groups were determined using the student’s *t*-test (*p < 0.05, **=p < 0.01, ***=p < 0.001). (Sp.PI = Sporozoite post-infection).

To study the immune responses against the pre-erythrocytic liver stage we analyzed the changes in B & T lymphocytes, and macrophages populations in HMNCs (from control and sporozoite infected mice groups) using flow cytometry-based dot plots (Fig. 2). Our results showed that the CD4^+^ T cell population (helper T cell) in HMNCs did not show any marked changes when compared to the sporozoite infected group to the control group. The percentage of CD4^+^ T cell population in the sporozoite infected group at 20 h and 40 h post sporozoite inoculation was 20.9 ± 0.5% and 18.6 ± 0.5% as compared to control group (20.1 ± 0.5%). Interestingly, the population of cytotoxic CD8^+^ T cells was significantly declined over time compared to control. Cytotoxic CD8^+^ T cells population in the control group was recorded 37.9 ± 0.6%, in comparison to sporozoite infected group where it was decreased to 27.7 ± 0.6% at 20 h, and 17.9 ± 0.6% at 40 h post sporozoite inoculation (Fig. 2). The CD19^+^ (B cell) population was also decreased in sporozoite infected group at both time points as compared to the control group. In the sporozoite infected group, the CD19^+^ population was 22.2 ± 0.8% and 22.8 ± 0.4% at 20 h and 40 h respectively, whereas, 25.8 ± 0.4% in the control group. Notably, we also observed a substantial decline in the macrophage population which was recorded 6.2 ± 0.6% in the control group whereas 3.9 ± 0.3% and 3.3 ± 0.4% in sporozoite infected group at 20 h and 40 h post sporozoite infected group (Fig. 2).

#### Assessment of T, B lymphocytes and macrophages in the spleen

**Fig. 3:**
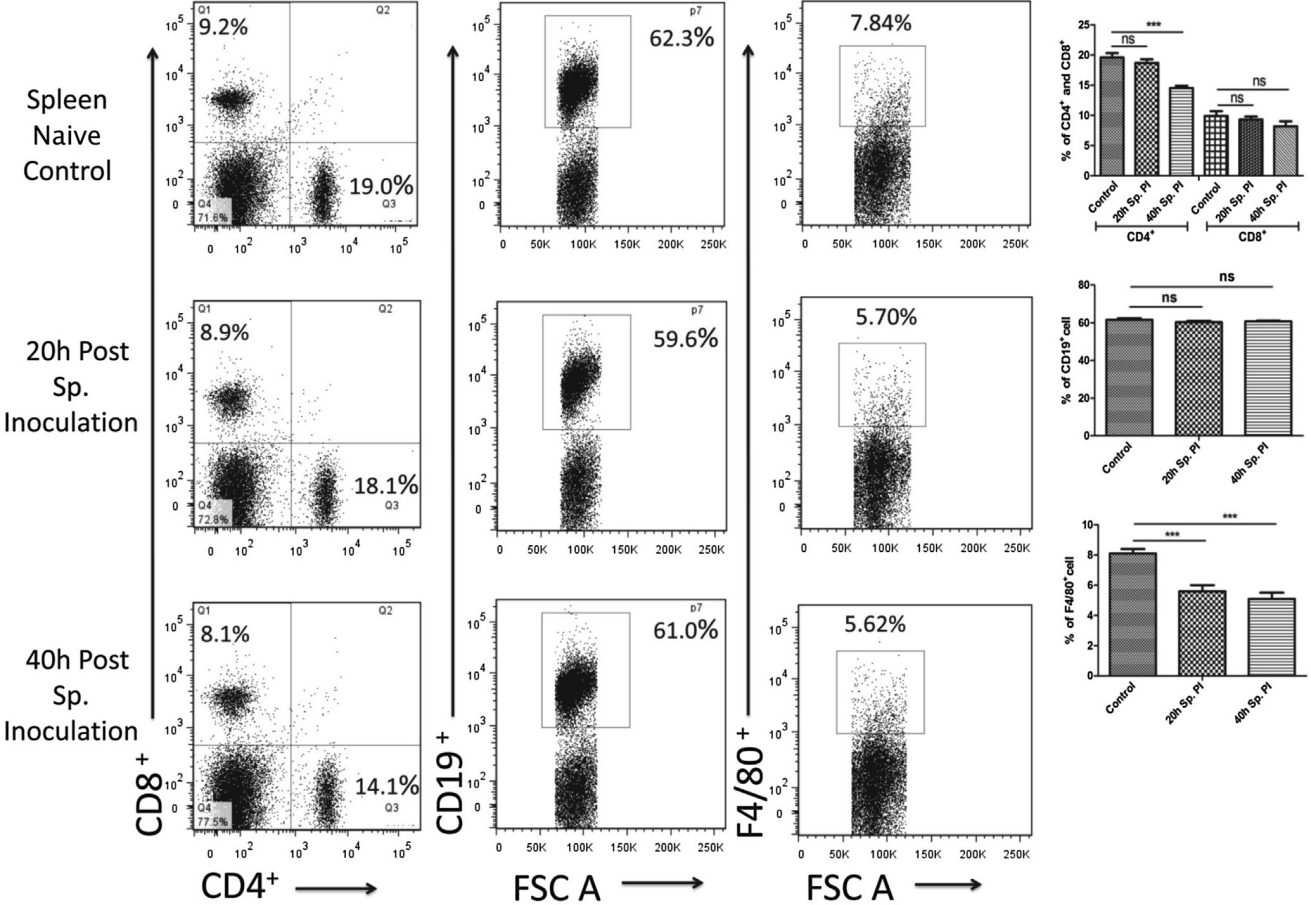
Phenotypic analysis of T cell, B cell and macrophage populations in spleen cells after *P. yoelii* sporozoite induced infection in mice. Percentage CD4^+^ and CD8^+^ population were determined in spleen cells isolated from different groups (naive and Sp. infected mice) using flow cytometry. Dot plots show representative images from one experiment and show percentage population of CD4^+^, CD8^+^, CD19^+,^ and F4/80^+^ cell populations, while the bar graph shows the Mean ± SD percentage population (n = 6). Statistical significances between different groups were determined using student’s *t*-test (*p < 0.05, **=p < 0.01, ***=p < 0.001). (Sp.PI = Sporozoite post-infection).

Similar to the liver, we also analyzed T & B cell and macrophage populations in the spleen of mice from control and sporozoite infected groups (Fig. 3). In spleen, the CD4^+^ T cell population is almost similar in control (19.6 ± 0.7%) and 20 h post sporozoite infection group (18.7 ± 0.7%). However, there is a decrement in the CD4^+^ T cell population in 40 h post sporozoite infected group (14.5 ± 0.4) when compared to control. In contrast, CD8^+^ T cell population did not altered as observed from the mean value for control group (9.9 ± 0.8%) and sporozoite infected group at 20 h (9.3 ± 0.5%) and 40 h (8.2 ± 0.4%) post sporozoite infection. Similarly, the CD19^+^ B cell population also remained unaltered in sporozoite infected group at both time points (60.3 ± 0.7% and 60.7 ± 0.4%) when compared to the control group (61.6 ± 0.8%), indicating no change in B cell response. Interestingly, the macrophage population was dramatically decreased post sporozoite infection in spleen cells. For control group macrophage population was 8.1 ± 0.3% (F4/80^+^) as compared with 20 h (5.6 ± 0.4%) and 40 h (5.1 ± 0.4%) post sporozoites infected group (Fig. 3).

#### Assessment of DCs in HMNCs population

**Fig. 4:**
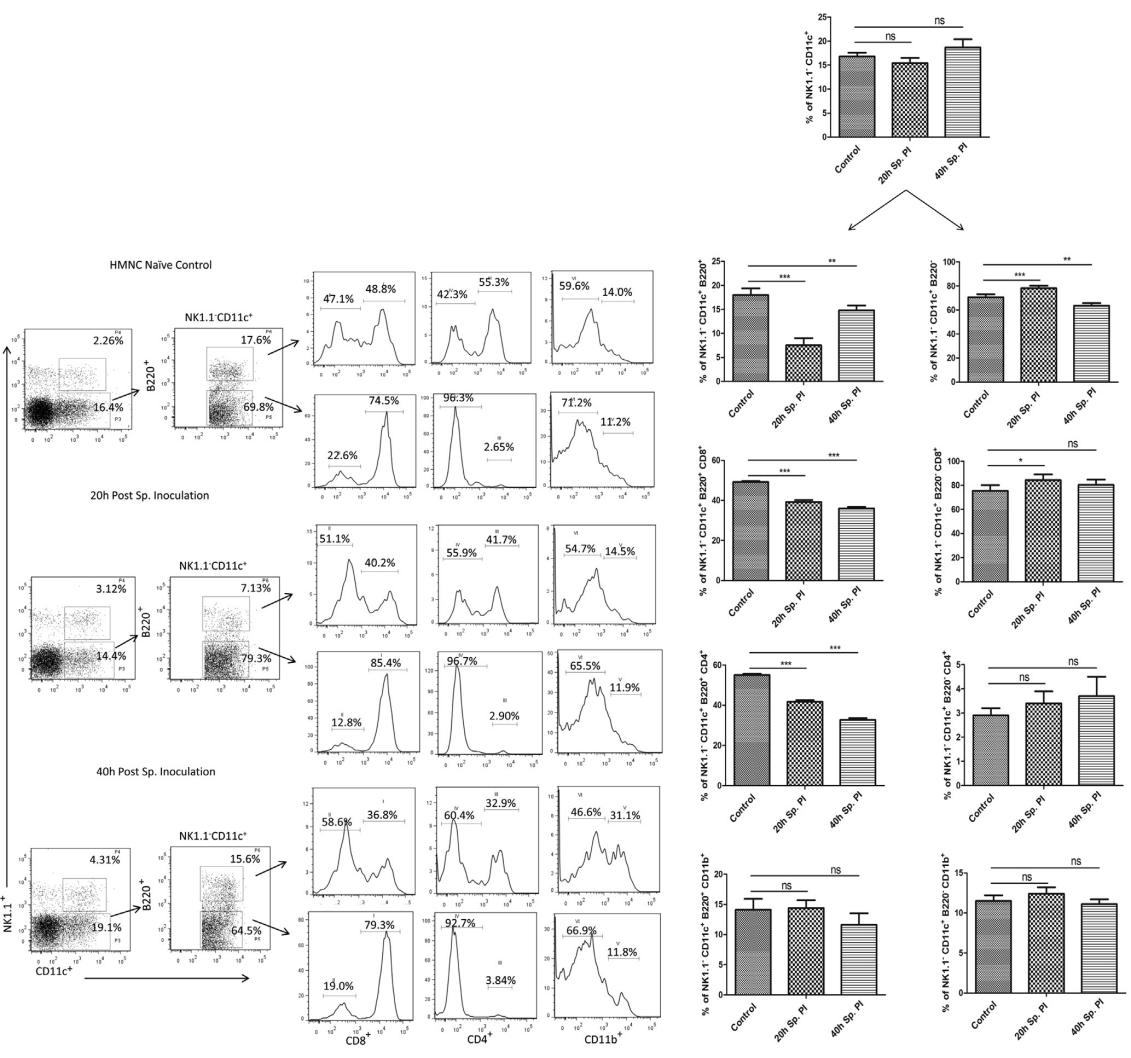
Phenotypic analysis of dentritic cells (DC) and DCs subset populations in HMNC after *P. yoelii* sporozoite induced infection in mice. Percentage NK1.1^−^ CD11c^+^ B220^+^ CD8^+^, NK1.1^−^ CD11c^+^ B220^+^ CD8^−^, NK1.1^−^ CD11c^+^ B220^+^ CD4^+^, NK1.1^−^ CD11c^+^ B220^+^ CD4^−^, NK1.1^−^ CD11c^+^ B220^+^ CD11b^+^ and NK1.1^−^ CD11c^+^ B220^+^ CD11b^−^ DCs subset population was determined in HMNCs isolated from different groups (naive and Sp. infected mice) using flow cytometry. Dot plots show representative images from one experiment, while bar graph shows Mean ± SD percentage population (n = 6). Statistical significances between different groups were determined using student’s *t*-test (*p < 0.05, **=p < 0.01, ***=p < 0.001). (Sp.PI = Sporozoite post-infection).

The phenotypic analysis of DC and DC subset populations in HMNC during the pre-erythrocytic stage was performed using various DCs surface markers. First, we marked the NK1.1^−^ CD11c^+^ DCs based on CDC11c expression and subsequently categorized this population into three subpopulations, namely plasmacytoid DCs (B220), lymphoid DCs (CD8 & CD4), and myeloid DCs (CD11b). This analysis shows that the percentage of DCs with CD11c^+^ marker was not markedly altered at 20 h and 40 h post sporozoite inoculation compared to the control group. Next, we observed two subpopulations of NK1.1^−^ CD11c^+^ HMNDC in naive mice samples representing plasmacytoid DCs [18.0 ± 1.4% (B220^+^)] and representing lymphoid and myeloid DCs [70.6 ± 2.5% (B220^−^)]. When we compared this result with the post sporozoite infected mice groups (20 h and 40 h), we observed a significant decline in plasmacytoid DCs at 20 h (7.5 ± 1.5%), which became regain to 14.8 ± 1.0% at 40 h (Fig. 4). Furthermore, plasmacytoid DCs (CD11c^+^ B220^+^) subsets with CD8^+^ and CD4^+^ surface markers were significantly decreased in sporozoite infected group at 20 h (39.2 ± 1.0 and 41.7 ± 0.8) and 40 h (36.0 ± 0.8 and 32.7 ± 0.9), when compared to the control group (49.2 ± 0.4 and 55.1 ± 0.6). However, DCs subset with NK1.1^−^ CD11c^+^ B220^+^ CD11b^+^ marker did not show any change between control uninfected and post sporozoite infected group (20 h and 40 h) (Fig. 4). Likewise, the sub-lineages of the lymphoid and myeloid DC (NK1.1^−^ CD11c^+^ B220^−^) subsets revealed a marginal surge in the cell population with CD8^+^ marker at 20 h and 40 h while subsets carrying CD4^+^ or CD11b^+^ marker were not markedly altered (Fig. 4).

#### Assessment of DCs in spleen cells population

**Fig. 5:**
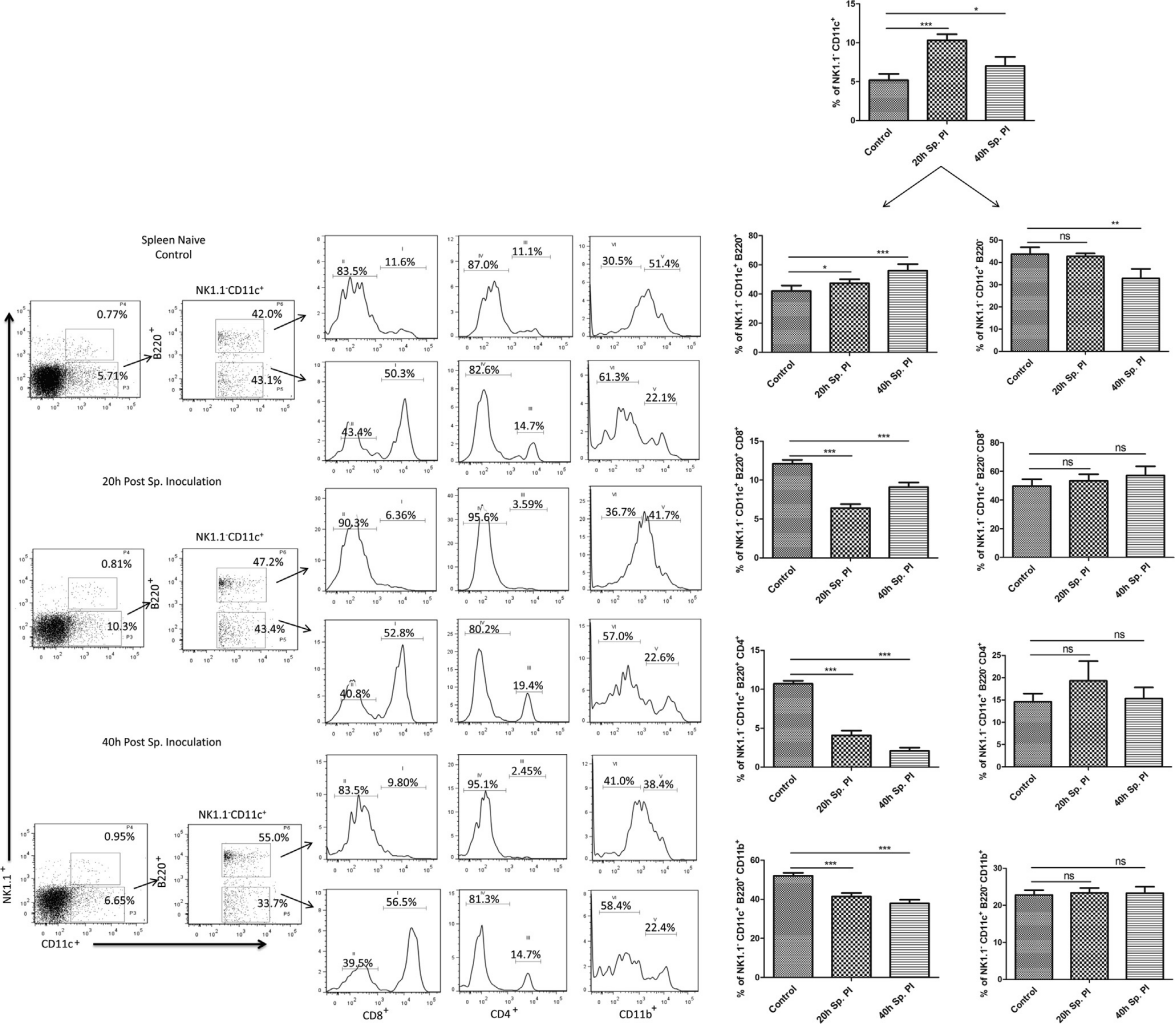
Phenotypic analysis of DC and DC subset populations in spleen during PE stage development after *P. yoelii* sporozoite induced infection in mice. Percent NK1.1^−^ CD11c^+^ B220^+^ CD8^+^, NK1.1^−^ CD11c^+^ B220^+^ CD8^−^, NK1.1^−^ CD11c^+^ B220^+^ CD4^+^, NK1.1^−^ CD11c^+^ B220^+^ CD4^−^, NK1.1^−^ CD11c^+^ B220^+^ CD11b^+^ and NK1.1^−^ CD11c^+^ B220^+^ CD11b^−^ DCs subset population was determined in splenic cells isolated from different groups (naive and Sp. infected mice) using flow cytometry. Dot plots show representative images from one experiment, while bar graph shows Mean ± SD percentage population (n = 6). Statistical significances between different groups were determined using student’s *t*-test (*p < 0.05, **=p < 0.01, ***=p < 0.001). (Sp.PI = Sporozoite post-infection).

Similar to HMNCc, spleen cell populations were also analyzed for assessment of DCs and DC subpopulations to compare the profile in naive control and sporozoite inoculated mice in spleen cell populations. Our results showed a steady increase in the percentage of spleen NK1.1^−^ CD11c^+^ DC population in 20 (10.3 ± 0.8%) and 40 h (7.0 ± 1.2%) post sporozoite infected group, when compared to control (5.2 ± 0.8%) (Fig. 5). Similarly, the population of plasmacytoid DCs was also increased in 20 h (47.3 ± 2.0%) and 40 h (56.0 ± 1.0%) post sporozoite infected group, when compared to control (42.0 ± 1.9%) (Fig. 5). The population showing B220^−^ expression (amongst NK1.1^−^ CD11c^+^ spleen DCs) was similar in control naïve (43.7 ± 0.6) and in 20 h (42.7 ± 0.7) post sporozoite infected groups, while 40 h (32.8 ± 0.9) post sporozoite infected group showed marked decrease in NK1.1^−^ CD11c^+^ B220^−^ spleen DC population. Kinetics of different subsets of plasmacytoid DCs in the spleen following sporozoite infection showed that plasmacytoid DCs subsets with CD8^+^ and CD4^+^ surface markers were significantly decreased in sporozoite infected group at 20 h and 40 h when compared to the control group. Likewise, spleen DCs subset with NK1.1^−^ CD11c^+^ B220^+^ CD11b^+^ also showed a significant reduction at 20 h (41.4 ± 0.9) and 40 h (37.9 ± 0.6) compared to control (52.0 ± 0.6). However, the analysis of sub-lineages of the lymphoid and myeloid DC (NK1.1^−^ CD11c^+^ B220^−^) subsets revealed that there was a marginal increase in the cell population with CD8^+^ and CD4^+^ marker at 20 h and 40 h, while subsets carrying CD11b^+^ marker was not altered noticeably.

### mRNA expression of pro-inflammatory cytokines

**Fig. 6:**
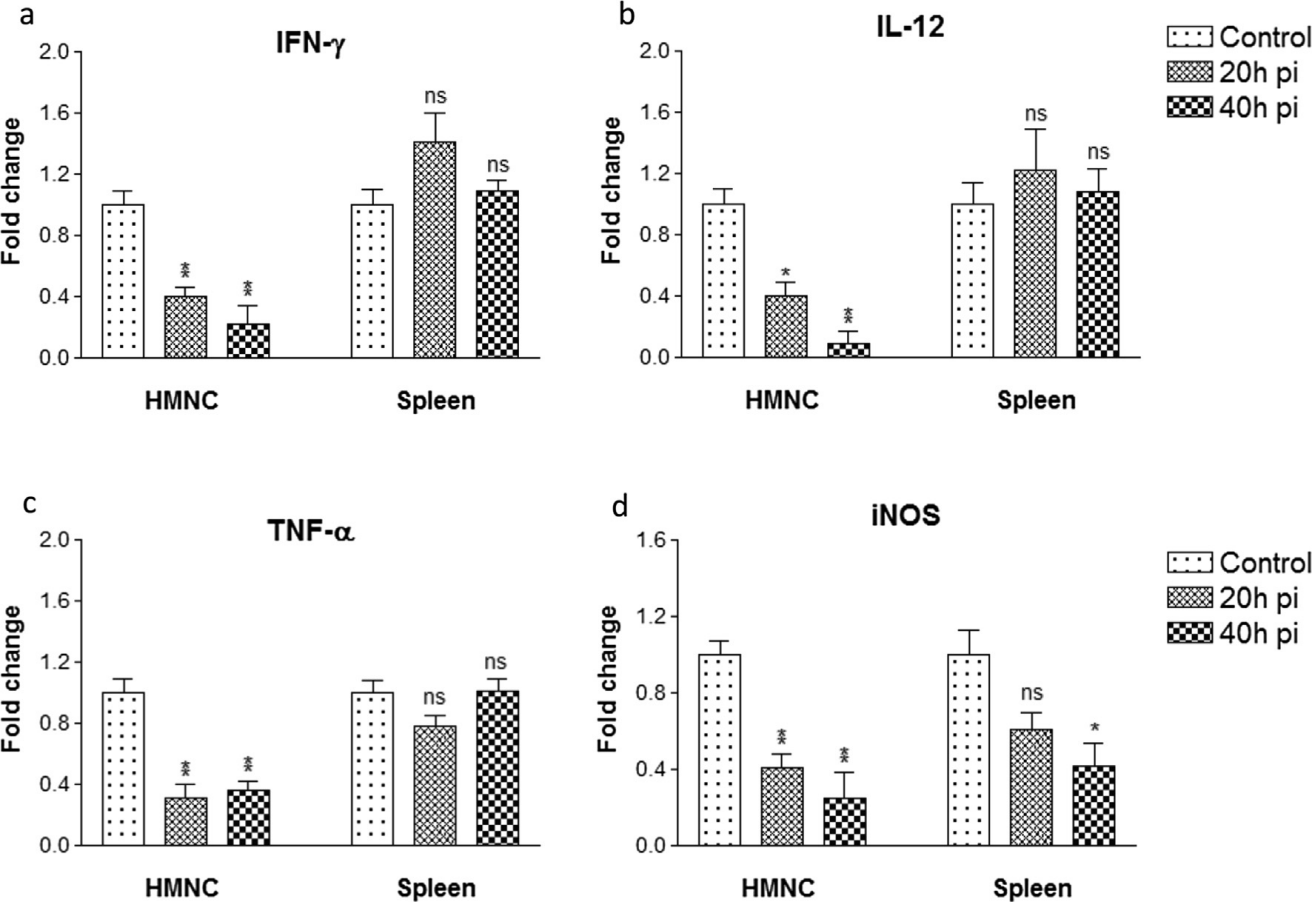
Relative mRNA expression of pro-inflammatory cytokines in HMNCs and spleen cells. HMNCs and spleen cells were isolated from control uninfected and *P. yoelii* sporozoites infected mice at different time points i.e. 20 h and 40 h. Each bar represents the Mean ± SD (n = 6) fold changes of respective cytokines (a) IFN-γ, (b) IL-12, (c) TNF-α, and (d) iNOS in HMNCs and splenocytes. Statistical significances between different groups were determined using student’s *t*-test (*p < 0.05, **=p < 0.01, ***=p < 0.001). (pi = post-sporozoite inoculation).

Following the phenotypic characterization of HMNCs and spleen cells, next, we measured the mRNA expression level of pro-inflammatory cytokines (i.e. IFN-γ, TNF-α, IL-12, and iNOS). After sporozoite inoculation, the mRNA expression of IFN-γ, and IL-12 in HMNCs was significantly down-regulated by ~2.5 folds to 5 folds after 20 h and 40 h post sporozoite infection in comparison to control uninfected HMNCs (Fig. 6a and b). Similarly, the mRNA expression level of TNF-α in HMNCs from the infected group was also down-regulated by 2.5 fold at 20 h and 40 h compared to uninfected control (Fig. 6c). However, in spleen cells, the IFN-γ and IL-12 mRNA expression levels were slightly increased to 1.4 and 1.2 fold at 20 h post sporozoite infection that reverts back to control at 40 h post sporozoite infection (Fig. 6a and b). In contrast, the mRNA expression of TNF-α in spleen cells was not changed significantly at 20 h and 40 h post sporozoite infection compared to control (Fig. 6c). Next, we measured the iNOS mRNA expression level in HMNC’s and spleen cells from control and sporozoite infected groups. As expected, we observed a significant down-regulation of iNOS mRNA expression level in a time-dependent manner post sporozoite infection (Fig. 6d). The iNOS mRNA expression level in HMNC’s from sporozoite infected group decreased to ~2.5 fold at 20 h which was further down-regulated to ~5 fold at 40 h as compared to the control group. Similarly, the mRNA expression of iNOS in spleen cells was also down-regulated to 0.6 ± 0.2 at 20 h and by 0.4 ± 0.2 at 40 h (Fig. 6d). Collectively, our results showed a significant down-regulation of pro-inflammatory cytokines in HMNC’s after sporozoite inoculation, however, the changes in spleen cells were non-significant except for iNOS expression.

### mRNA expression of anti-inflammatory cytokines

**Fig. 7:**
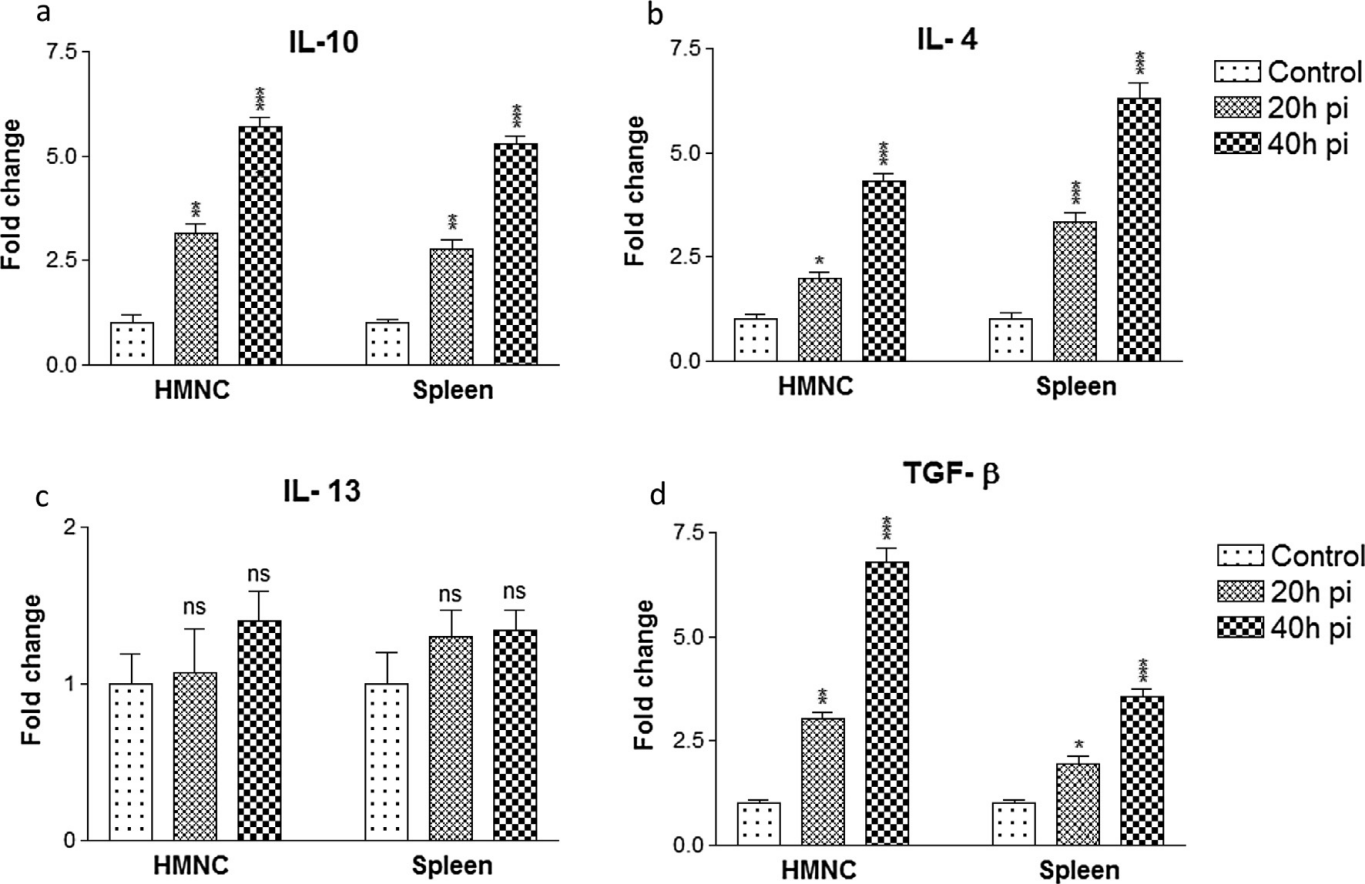
Relative mRNA expression of anti-inflammatory cytokines in HMNCs and spleen cells. HMNCs and spleen cells were isolated from control uninfected and *P. yoelii* sporozoites infected mice at different time points i.e. 20 h and 40 h. Each bar represents the Mean ± SD fold changes of respective cytokines (a) IL-10, (b) IL-4, (c) IL-13 and (d)TGF-β in HMNCs and splenocytes. Statistical significances between different groups were determined using student’s *t*-test (*p < 0.05, **=p < 0.01, ***=p < 0.001). (pi = post-sporozoite inoculation).

mRNA expression profiling of anti-inflammatory cytokines such as IL-10, IL-4, IL-13, and TGF-β revealed a significant upregulation post sporozoite infection in the HMNcs and spleen cells. Our results suggested that after sporozoite inoculation IL-10 mRNA expression was highly significantly upregulated in HMNC’s with 3.1 ± 0.4 folds and 5.6 ± 0.4 folds at 20 h and 40 h sporozoite inoculation. Likewise, splenic cells also showed significant up-regulation of IL-10 mRNA expression by 2.8 ± 0.3 folds at 20 h, which was further up-regulated to 5.3 ± 0.3 folds at 40 h (Fig. 7a). Similarly, the mRNA expression of IL-4 in HMNC’s from the infected group was also up-regulated by 1.9 ± 0.3 folds at 20 h and 4.3 ± 0.3 folds at 40 h. In spleen cells, the mRNA expression level of IL-4 was up-regulated by 3.3 ± 0.4 folds at 20 h and 6.3 ± 0.6 folds at 40 h in sporozoite infected group (Fig. 7b). However, IL-13 mRNA expression remains almost similar in HMNC’s and spleen cells from the infected group at 20 h and 40 h post sporozoite infection as compared to control uninfected group (Fig. 7c). In HMNCs, the mRNA expression level of TGF-β was upregulated to 3.0 ± 0.3 folds at 20 h and 6.8 ± 0.6 folds at 40 h in sporozoite infected group. Similarly, in spleen cells, the mRNA expression of TGF-β was up-regulated by 1.9 ± 0.3 folds at 20 h and 3.5 ± 0.3 folds at 40 h in sporozoite infected group as compared to control group (Fig. 7d). Altogether, our results showed that anti-inflammatory cytokines such as IL-10, IL-4 and TGF-β showed marked upregulation in HMNCs and spleen cells in the post sporozoite infected group.

### Dynamics of pro and anti-inflammatory cytokines level in culture supernatants of HMNCs and spleen cells

**Fig. 8:**
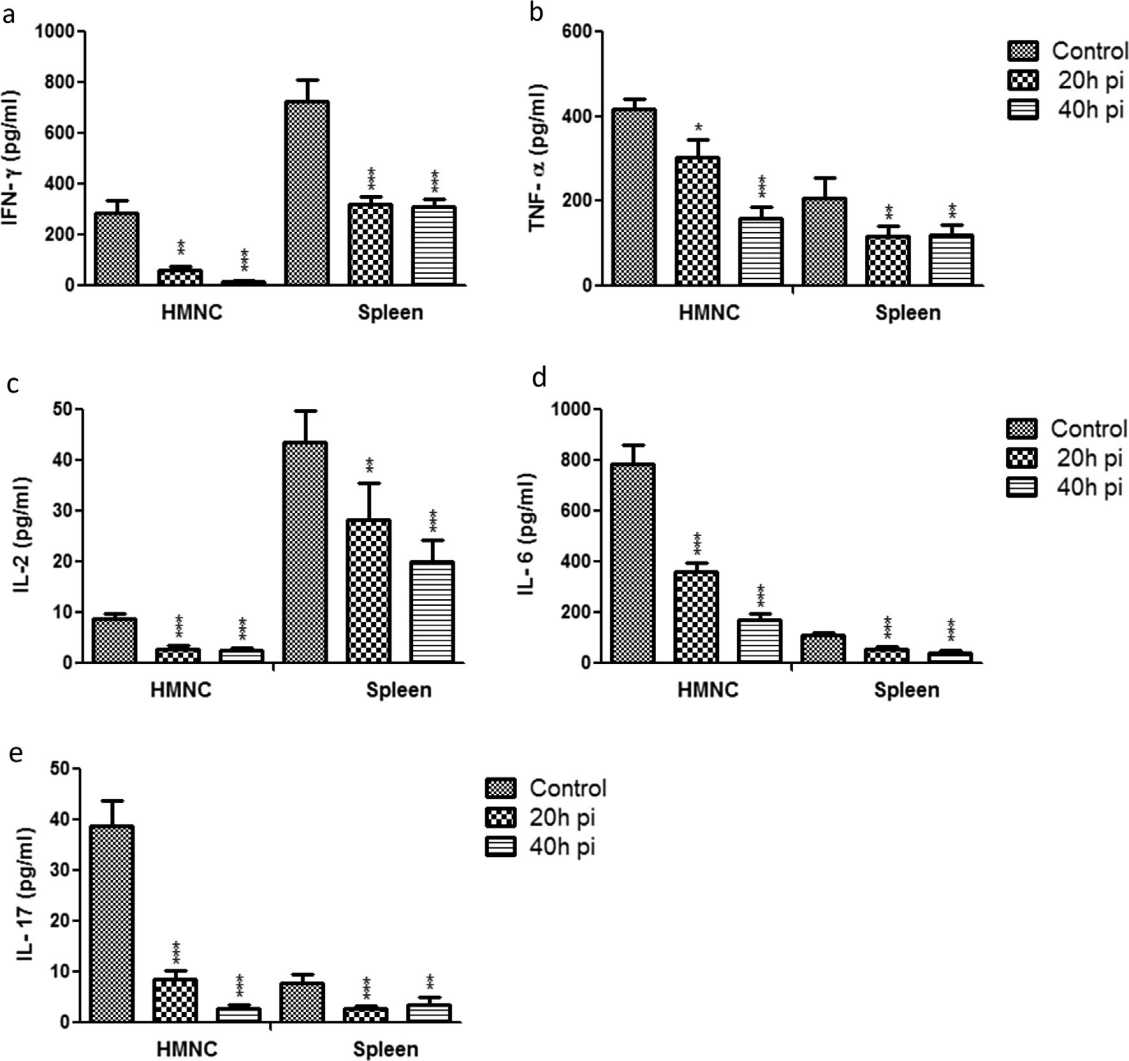
Measurement of pro-inflammatory cytokines in culture supernatants of HMNCs and spleen cells. HMNCs and spleen cells were isolated from control uninfected and *P. yoelii* sporozoites infected mice at different time points (i.e. 20 h and 40 h). The cells were seeded into 48-well tissue culture plates, culture and stimulated with either lipopolysaccharide (LPS) (1 µg/well). Subsequently, supernatants were collected from LPS stimulated cells and used for cytokines estimation. Each bar graph shows concentration (pg/ml) of the respective cytokines (a) IFN-γ, (b)TNF-α, (c) IL-2, (d) IL-6, and (e) IL-17. Statistical significances between different groups were determined using student’s *t*-test (*p < 0.05, **=p < 0.01, ***=p < 0.001). (pi = post-sporozoite inoculation).

**Fig. 9:**
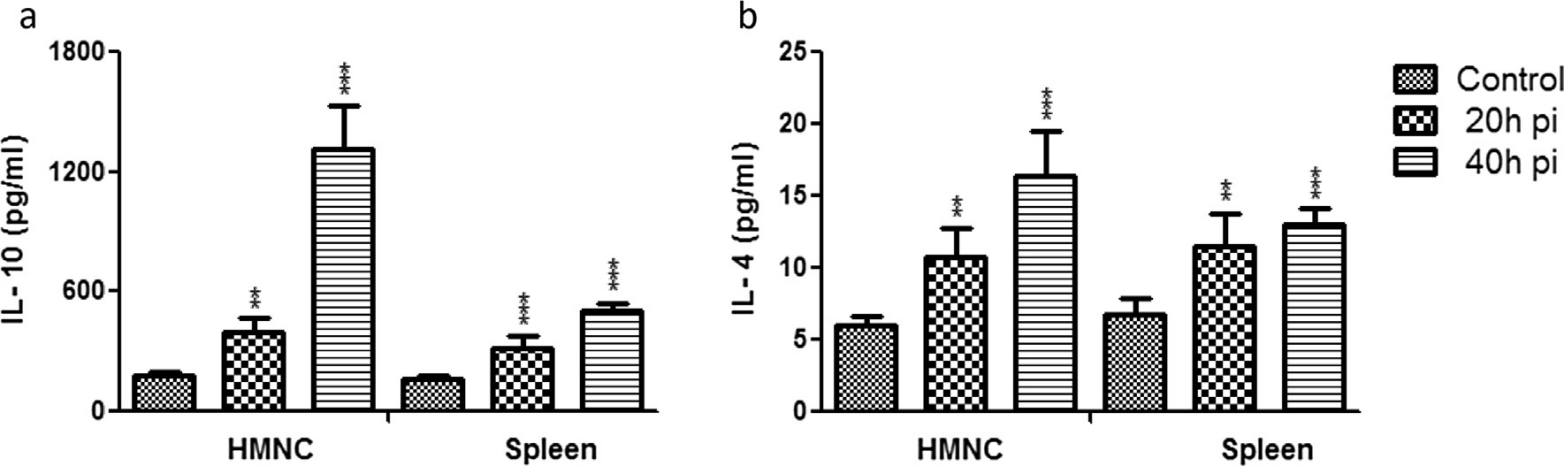
Measurement of anti-inflammatory cytokine level in culture supernatants of HMNCs and spleen cells. HMNCs and spleen cells were isolated from control uninfected and *P. yoelii* sporozoites infected mice at different time points (i.e. 20 h and 40 h). The cells were seeded into 48-well tissue culture plates, culture and stimulated with either lipopolysaccharide (LPS) (1 µg/well). Subsequently, supernatants were collected from LPS stimulated cells and used for cytokines estimation. Each bar graph shows the Mean ± SD concentration (pg/ml) of the respective cytokines (a) IL-10 and (b) IL-4. Statistical significance between different groups were determined using student’s *t*-test (*p < 0.05, **=p < 0.01, ***=p < 0.001). (pi = post-sporozoite inoculation).

Since we noticed significant changes in pro and anti-inflammatory cytokines mRNA levels in HMNCs and spleen cells from sporozoite infected groups. Therefore, next, we measured directly the concentration of pro and anti-inflammatory cytokines level in culture supernatants of HMNCs and spleen cells. Our results showed that culture supernatant of HMNCs isolated from sporozoite infected groups (20 h and 40 h) showed a significantly lower level of IFN-γ, TNF-α, IL-2, IL-6 and IL-17 in comparison to control uninfected group (Fig. 8a–e). The 20 h post sporozoite infected group exhibited lower levels of pro-inflammatory cytokines i.e. IFN-γ, TNF-α, IL-2, IL-6 and IL-17, which was further reduced in 40 h post sporozoite infected group, in contrast, to control (Fig. 8). These results were in accordance with mRNA expression profiling of pro-inflammatory cytokines as obtained from HMNCs. However, in contrast to mRNA profiling, we observed a significant reduction in pro-inflammatory cytokines levels from the culture supernatant of splenic cells in sporozoite infected groups as compared to the control group (Fig. 8). Furthermore, we also observed that the level of anti-inflammatory cytokines IL-10 and IL-4 was significantly elevated in the culture supernatant of HMNCs from 20 h to 40 h post sporozoite infected as compared to control uninfected group (Fig. 9). A similar observation was also noted for anti-inflammatory cytokines IL-10 and IL-4 in the culture supernatant of splenic cells from sporozoite infected groups as compared to control uninfected group (Fig. 9).

### NO generation in culture supernatants of HMNCs and spleen cells

**Fig. 10:**
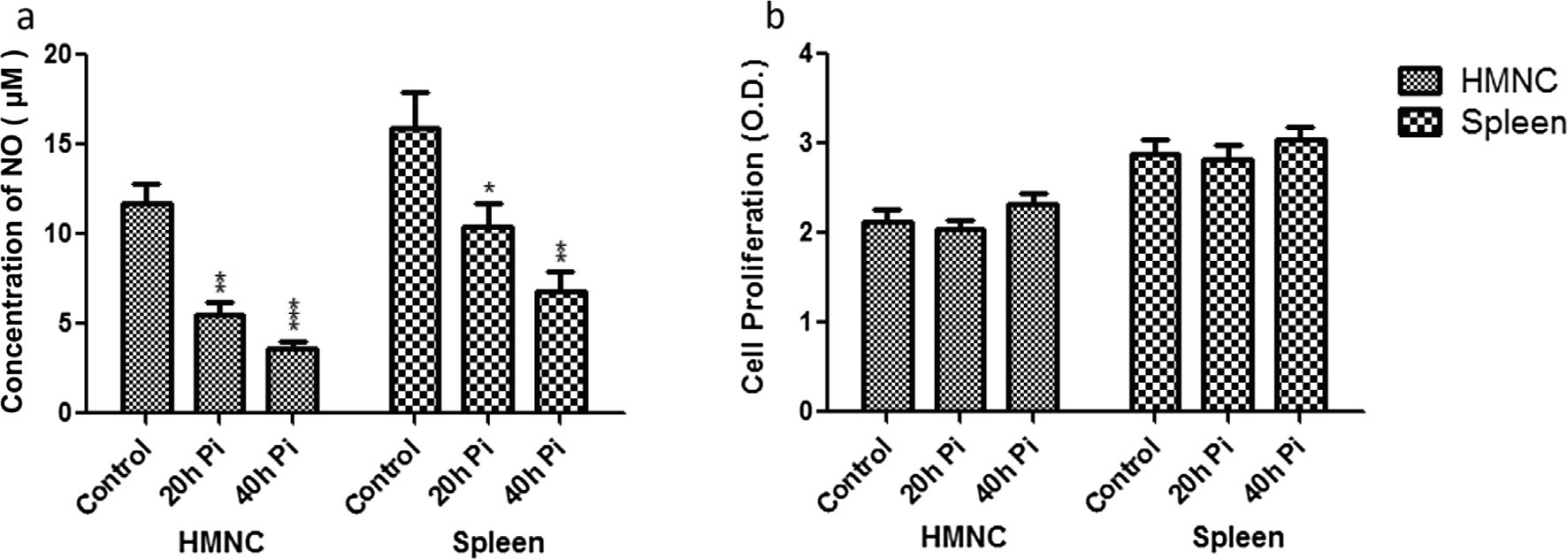
Nitric oxide (NO) and cell proliferation response in HMNCs and splenocytes. (a) Nitric oxide released in culture supernatants of hepatic and splenic macrophages from control uninfected and *P. yoelii* sporozoites infected mice at different time points (i.e. 20 h and 40 h) was determined by Griess reagent. (b) Cell proliferation response in HMNcs and spleen cells from control uninfected and *P. yoelii* sporozoites infected mice at different time points (i.e. 20 h and 40 h). Absorbance was determined at 450 nm using ELISA reader. Each bar graph shows the Mean ± SD values from different groups. (pi = post-sporozoite inoculation).

To determine a causal relationship between cytokines expression and NO release, next, we checked the NO level in the HMNCs and spleen cells culture supernatants from control uninfected and sporozoite infected group (at 20 h and 40 h post sporozoite infection) (Fig. 10a). Our results showed a time-dependent reduction in NO level following sporozoite inoculation. The NO level in HMNCs culture supernatant from the control group was 11.7 ± 1.2 µM, whereas it declines to 5.6 ± 0.6 µM and 3.6 ± 0.4 µM at 20 h and 40 h post sporozoite infected groups, respectively. Similarly, in the culture supernatant of spleen cells, NO concentration was also declined in sporozoite infected group to 10.4 ± 1.2 µM and 6.8 ± 1.1 µM at 20 and 40 h post sporozoite inoculation as compared to control group (15.9 ± 2.1 µM) (Fig. 10a). These results suggesting that the downregulation of proinflammatory cytokines and upregulation of anti-inflammatory cytokines following the progression of pre-erythrocytic liver stage parasite results in reduced NO release that may require for the establishment of liver stage development.

### Cell proliferation response in HMNCs and spleen cell

Cell proliferation of liver HMNCs and spleen cells in control and sporozoites infected mice was assessed by XXT assay and the results plotted as optical density (OD) versus time (Fig. 10b). Our results of XTT assay showed that both HMNCs and spleen cells exhibited similar proliferative response in control uninfected mice and 20 h and 40 h post sporozoite infected mice. Thus, these data show that the progression of sporozoite infection does not have any effect on the cell proliferation rate in the liver and spleen (Fig. 10b).

## Discussion

The liver stage, also known as the pre-erythrocytic stage is relatively short and asymptomatic. During the liver stage, a relatively low number of parasites provide an advantage to the immune system to eliminate infected hepatocytes and thereby preventing parasite transition into the blood-stage [24]. However, malaria parasites have continuously evolved and acquire diverse immune evasion mechanisms that result in poor immune responses [25]. Despite the years of laboratory and clinical research, we are still far away from an effective long-lasting preventative vaccine for malaria. Most of the studies performed on liver-stage mainly deal with the adaptive immune responses induced by immunization with irradiated sporozoites [26]. However, protective immune responses during primary infection remain elusive [27]. In this view, in the present study, we aimed to investigate the immune responses against sporozoite induced primary infection and explored the dynamics of various immune cell populations and cytokine response in liver and spleen following *P. yoelii* sporozoites infection in Swiss albino mice model. *P. yoelii* is the widely used rodent malaria parasite to study the pathology of *Plasmodium* species. It is also an acceptable model for pre-erythrocytic stage development and extensively used in studies dealing with the potency of novel vaccine or drug candidates [28].

To initiate our study, we first determined the liver stage parasite load and blood-stage patency following post sporozoite inoculation to distinguish between the pre-erythrocytic and erythrocytic stage parasites. For this purpose, we measured the parasite load in the liver using quantitative RT-PCR based detection of 18S rRNA copy number. At the same time, we also checked blood-stage patency in mice infected with the same number of sporozoite to find the appropriate time point to clearly distinguish the stage of the liver and blood. Our results validated that the sporozoites used in this study were infective/ viable and at an initial 20 h and 40 h post sporozoite infection only pre-erythrocytic stage present, while blood-stage developed later on. These results were in close agreement with previous studies which suggested that in mouse model pre-erythrocytic stage last ~50 h post sporozoite inoculation [22], [23]. Based on these observations, we choose 20 h and 40 h post sporozoite infection time points for further study to analyze the cellular immune responses elicited by pre-erythrocytic liver stage parasite in HMNCs and splenic cells***.*** In our immune-phenotypic analysis*,* we observed a significant reduction in the CD8^+^ T, CD19^+^ B and macrophage cell population in the liver (HMNCs) from the sporozoite infected group (i.e. 20 h and 40 h psi) compared to control uninfected group. CD8^+^ T cells are a vital subpopulation of MHC class I-restricted T cells and are mediators of adaptive immunity. These cells help in boosting high levels of antibodies during natural infection with the parasite and to kill the parasite-infected cells. Previous studies with mouse malaria model suggested an important role of CD8^+^ T cells in defense against sporozoite challenge [29], and liver-stage malaria [30]. Another important observation in our study is the reduced percentage of CD19^+^ B cells in the sporozoite infected group as compared to the control group. B cells or plasma cells play a vital role in humoral immunity and there are the evidential facts that suggest the impairment of functional B cell response in the absence of T cell and vice versa [31].

Likewise, we also observed a significant reduction in the antigen-presenting macrophages in the liver and spleen from the sporozoites infected groups. Modulation of the antigen presentation by major histocompatibility complex 1 (MHC I) on infected hepatocytes is thought to be an important strategy of the parasite to successfully evade the immune system and establish infection in hepatocytes [32]. Altogether, these results suggest that liver-stage parasites bypass the protective immunity by suppressing the production of effectors' immune cells and at the same time minimize the antigen presentation by reducing the number of hepatic macrophages. Furthermore, in our study, we also noted the reduced activity of DCs. The kinetics analysis of different DC subtypes in HMNCs and spleen cells suggests that plasmacytoid DCs are significantly decreased in the liver and spleen of mice following sporozoites infection. However, myeloid and lymphoid DCs population remains unaffected. Plasmacytoid DCs are the main producers of IFN-γ, a key mediator of inflammatory immune responses. Furthermore, it is well documented that IFN-γ plays a vital role in controlling Plasmodium infection in both the liver and blood stages of the malaria parasite [33]. This highlights the importance of plasmacytoid DCs in protective immunity against liver-stage parasite development. In our phenotypic characterization of immune cells, we noted a significant reduction in immune effectors cells including plasmacytoid DCs, therefore we next checked the downwards effect of the pre-erythrocyte parasite on cytokines profile. As expected, we observed a significant downregulation in the mRNA levels of pro-inflammatory cytokines (IFN-γ, IL-12, TNF-α, iNOS) and upregulation in the mRNA levels of anti-inflammatory cytokines (IL-10, IL-4) at 20 h and 40 h post sporozoite infection as compared to control. These results were further supported by direct cytokine measurement from the culture supernatant of HMNCs and spleen cells from sporozoite infected groups (20 h and 40 h), which revealed a significantly lower level of pro-inflammatory cytokines (IFN-γ, TNF-α, IL-2, IL-6, and IL-17) and high level of anti-inflammatory cytokines in comparison to control uninfected group. These results were in accordance with other studies which reported the impairment of pro-inflammatory cytokine response as a result of *Plasmodium* replication and development inside hepatocytes [34], [35]. Cytokine's response is linked to the variation in susceptibility of mice to different parasite strains [36].

The protective role of pro-inflammatory cytokines has been confirmed by several studies employing sporozoite immunization in mice model of malaria [34], [37]. It has been demonstrated that *in-vitro* treatment of infected hepatocytes with IFN-γ eliminates the *P. berghei* and *P. falciparum* parasites [13], [38], [39], [40], whereas, *in vivo* administration of IFN-γ partially protected against sporozoite challenge with *P. berghei* in mice and *P. cynomolgi* in monkeys model [41], [42]. Moreover, *in vivo* depletion of IFN-γ abrogated the protective immunity induced by *P. berghei* sporozoites in mice. As a response to infection, host inflammatory immune responses gets activated which results in phagocytosis, ROS generation and oxidative killing of pathogens [43]. Since, IFN-γ induces the production of NO *in vitro* and *in vivo* following *P. berghei*, *P. yoelii* or *P. falciparum* sporozoite infections [44]. We next checked the NO release from the culture supernatant of HMNCs and spleen cells from control and sporozoite infected mice. Intriguingly, we observed a time-dependent noticeable reduction in NO release from HMNCs and spleen cells of sporozoite infected groups (20 h and 40 h). This suggests that the sporozoites infection and subsequent development in the liver induce the anti-inflammatory immune responses and deactivating effect of the secondary messenger (cAMP) to modulate the kupffer cells function. This can be attributed due to the up-regulation of an anti-inflammatory enzyme, HO-1 (heme oxygenase-1) in hepatocytes as observed previously following *P. berghei* and *P. yoelli* sporozoites infection. The elevated level of HO-1 is believed to promote the development of the liver stages by counteracting the host inflammatory response [45], [46]. Overall, these finding suggests that during the pre-erythrocytic stage in mice, Th2 biased immune responses play an important role in parasite protection and survival in the liver. However, future studies are further needed to address several important and unresolved questions including (1) how parasite modulates the host immune system, (2) what are the host and parasite factors involved and, (3) the underlying molecular and cellular mechanisms.

## Conclusion

Different suppressive immune mechanisms used by the liver-stage malaria parasite to modulate host immune responses and to establish successful infection inside hepatocytes. In our study we found several important observations that explain immune evasion of intra-hepatic sporozoites and essentially includes: (1) modulation of protective immunity by suppressing immune effectors CD8^+^ T cells and CD19^+^ B cells population, (2) minimize the antigen presentation by curtailing the antigen-presenting macrophages and dendritic cells population and (3) by modulating the host inflammatory responses to aid the intra-hepatocyte parasite development i.e. decrease in pro-inflammatory and increase in anti-inflammatory cytokines level. Taken together, our data provide crucial information on immune responses generated during primary sporozoite infection in mice models. Based on these observations, we concluded that during liver stage development antigen presentation and protective immunity is suppressed and Th2 biased cytokine response dominate to assists parasite survival. Our study provides important insights from animal models of malaria immunity that can assist in creating novel strategies to develop better therapeutic options and effective vaccines to reduce this global health problem.

## Compliance with Ethics Requirements

*All Institutional and National Guidelines for the care and use of animals (fisheries) were followed.*

*Ethical approval (No: IAEC/2012/67-N) was obtained from CSIR-Central Drug Research Institute’s ‘Institutional Animal Care and Use Committee’ recognized by ‘Committee for the Purpose of Control and Supervision of Experiments on Animals (CPCSEA)’, Government of India to perform experiments on animals.*

## Declaration of Competing Interest

*The authors declare that they have no known competing financial interests or personal relationships that could have appeared to influence the work reported in this paper.*
